# Resolution of refractory perigraft seroma from Triplex graft after 14 months of percutaneous fluid drainage: a case report

**DOI:** 10.1186/s44215-025-00214-5

**Published:** 2025-07-01

**Authors:** Yukino Iijima, Takaya Hoashi, Koichi Toda, Akinori Hirano, Ryusuke Hosoda, Yuji Fuchigami, Takaaki Suzuki

**Affiliations:** 1 Department of Pediatric Cardiac Surgery, Saitama Medical University International Medical Center, Hidaka, Saitama Japan; 2 Department of Pediatric Cardiology, Saitama Medical University International Medical Center, Hidaka, Saitama Japan

**Keywords:** Perigraft seroma, Triplex vascular graft, Congenital heart disease

## Abstract

A 21-year-old female patient had undergone two open heart surgeries in childhood, including Konno ventriculoplasty with mechanical aortic valve replacement. She underwent a redo mechanical aortic valve replacement and patch enlargement of stenotic ascending aorta using a triplex prosthetic graft. Unfortunately, 1 month after surgery, the patient was readmitted to the hospital with a diagnosis of midline chest wound infection. A culture sample from the wound revealed Serratia marcescens, however, subsequent all culture tests were negative. Since then, there has been a continued serous discharge from the caudal side of the midline skin incision scar and chest tube removal scar in the chest. Despite the implementation of four open chest treatments, the issue of perigraft seroma persisted, and a diagnosis was ultimately made. During her subsequent admission, negative pressure wound therapy was employed, followed by daily sterilization and film dressing post-discharge. It is understood that a gradual decrease in drainage and complete resolution of the seroma occurred 21 months after surgery, without the removal of the implanted triplex patch.

## Background

It has been observed that perigraft seroma, a complication associated with aortic replacement surgery involving artificial grafts, occurs in some cases [1]. There is no consensus on a treatment strategy, and in some cases, treatment options are limited to the removal of the implanted artificial graft. In this case, we present a patient with refractory perigraft seroma following aortic surgery with a Triplex graft as a patch. The condition was successfully resolved without the removal of the implanted Triplex graft, through long-standing percutaneous fluid drainage following several ineffective surgical interventions.

## Case presentation

**Fig. 1:**
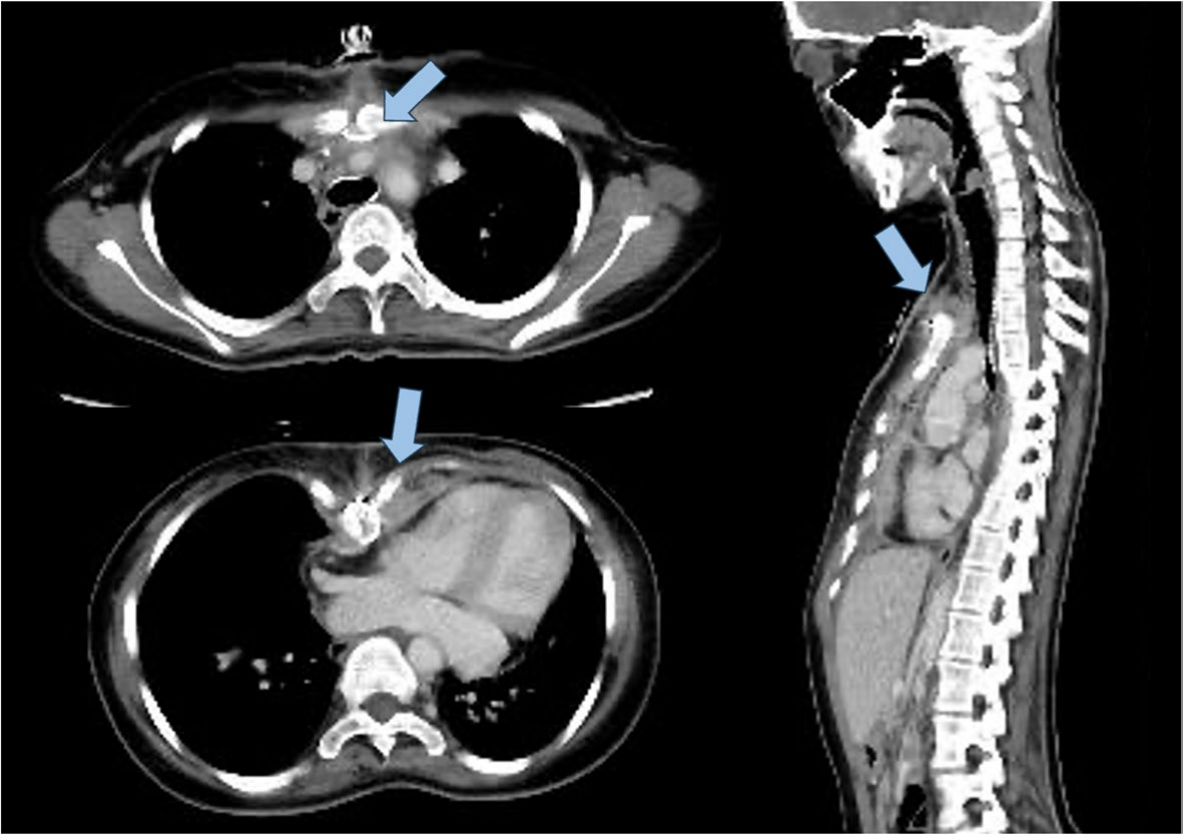
Computed tomography results (arrow; fluid collection)

It is understood that the 21-year-old female patient has a medical history that includes surgical procedures such as coarctation repair, closure of ventricular septal defect, and mechanical aortic valve replacement (AVR) with Konno ventriculoplasty during childhood. She subsequently underwent a redo mechanical AVR and a patch enlargement of the stenotic ascending aorta using a triplex prosthetic graft. She was discharged from the hospital 11 days after the surgery. However, approximately 1 month after the initial surgery, there were signs of wound dehiscence and exudate observed at the caudal side of the midline skin incision scar in her chest. She had no fever (body temperature 36.8 °C) and her blood test result was WBC 5980/dL, CRP 0.49, neutrophil 78.1%. Serratia marcescens was detected in the wound culture, which led to the commencement of antibiotic administration and wound irrigation. A subsequent enhanced computed tomography (CT) scan revealed the presence of a small amount of fluid collection located just below the sternum (Fig. 1), thereby raising suspicion of mediastinitis. In light of these findings, we opted to proceed with open sternal irrigation. However, the bacterial culture test of the fluid collected 3–4 cm from the top of the sternum proved negative. A Triplex patch, which was placed beneath the pericardial extended polytetrafluoroethylene (ePTFE) sheet, exhibited a near-complete absence of adhesion. It is also worth noting that all subsequent culture tests of the discharge or blood were negative.

After a period of 1 month, it was observed that there was a continued presence of serous discharge seeping from the caudal side of the midline skin incision scar in the chest. A second open sternal exploration was performed, but no causative agent for the discharge could be identified, including surgical site infection. Consequently, serous fluid leakage from the wound has persisted. Rather than an infection, it was suspected that the patient may have had a hypersensitivity reaction to the artificial materials or perigraft seroma, but the patient and her parent declined to replace the Triplex patch. Therefore, a third chest re-entry was performed to remove the ePTFE pericardial sheet and coat the Triplex patch with fibrin sealant glue and patch. However, this approach did not yield the desired outcome.

**Fig. 2:**
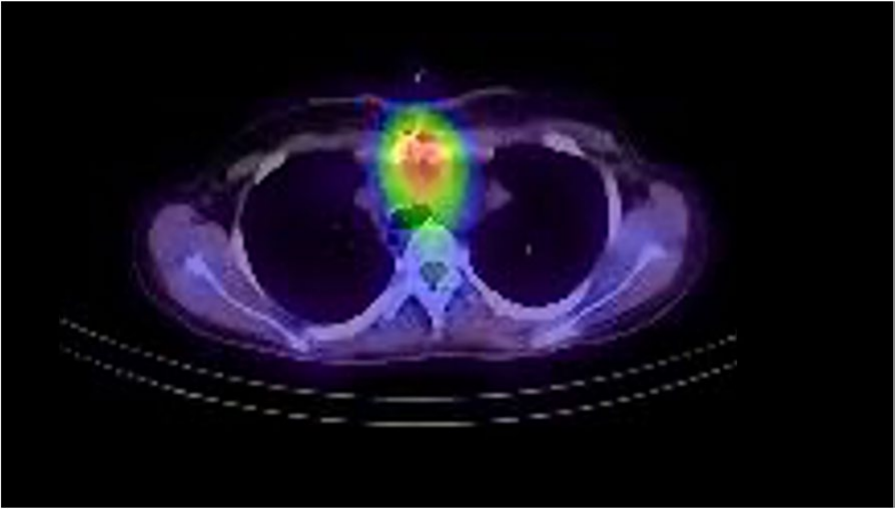
Gallium scintigraphy result

Subsequently, the severity of the discharge from the wound diminished, yet it persisted. The results of the gallium scintigraphy revealed a solitary site of nuclear uptake, exhibiting a sternal pattern, which manifested as the most pronounced area of uptake (Fig. 2). Given its concurrence with the position of the sternal wire, the possibility of osteomyelitis could not be disregarded. As a result, the most cephalad wire was carefully extracted, and the upper mediastinum was irrigated through a partially opened sternum.

**Fig. 3:**
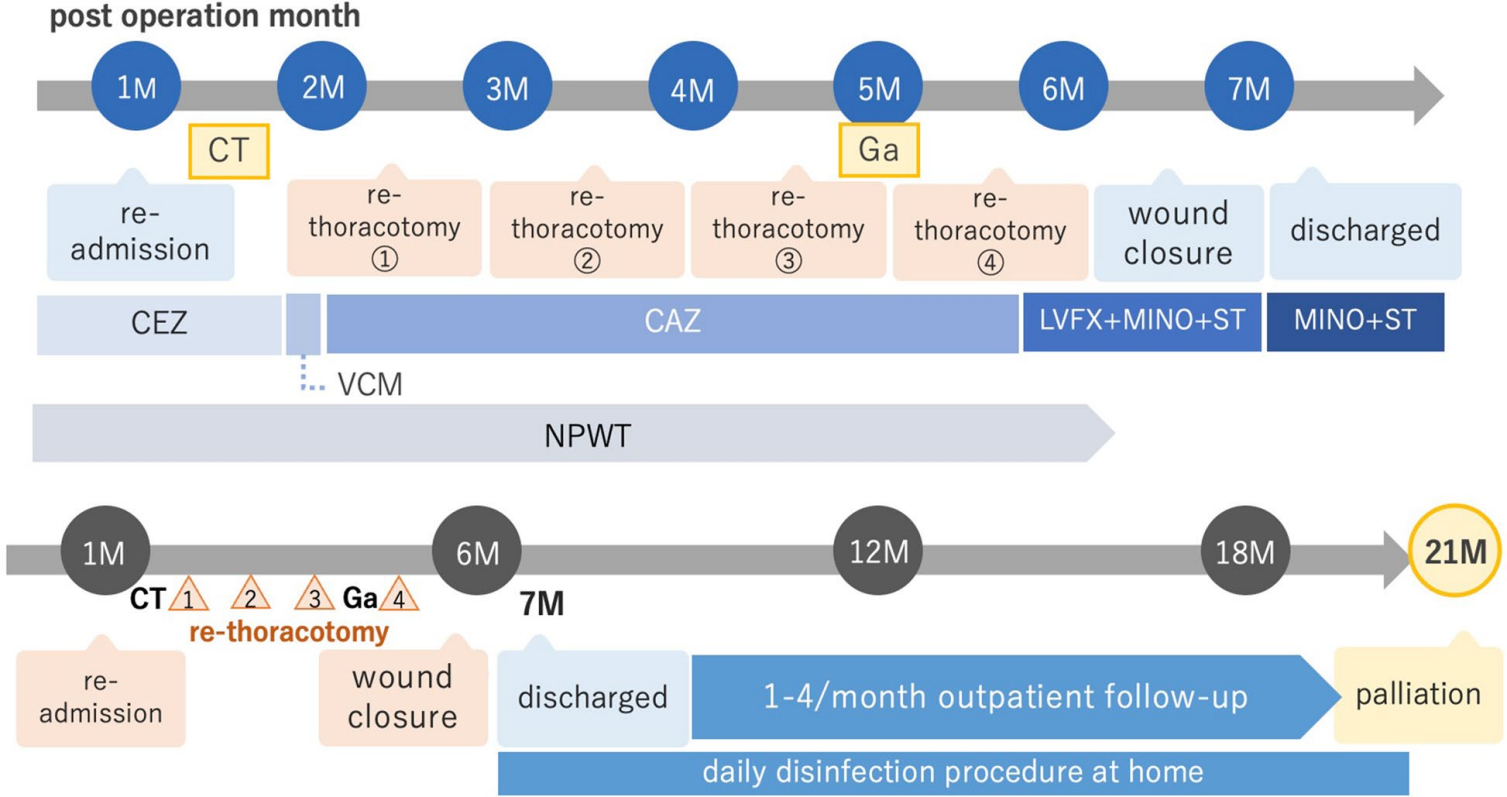
Clinical course of a patient (M post-operation month, CT computed tomography, Ga gallium scintigraphy, △ re-thoracotomy, NPWT negative pressure wound therapy, CEZ cefazolin, VCM vancomycin, CAZ ceftazidime, LVFX levofloxacin, MINO minocycline, ST sulfamethoxazole trimethoprim)

It is our understanding that the fluid drainage was localized, but it persisted after the aforementioned four times open-chest surgical treatment. Ultimately, perigraft seroma was diagnosed. Given that the duration of hospitalization had already extended to 7 months at that time, disinfection treatment was prescribed, and the patient was discharged (Fig. 3). During the 7 months of her prolonged hospitalization, no signs of infection such as fever, elevated C-reactive protein, or increased neutrophil had been observed, except for every few days following each surgical treatment.

The patient underwent continued regular outpatient follow-ups. Fourteen months after discharge and 21 months postoperatively, the fluid drainage was terminated and skin indentation was completely closed. Echocardiography revealed no fluid accumulation in the mediastinum or pericardium, and the patient was considered to be in remission.

## Discussion

It is important to note that the identification of perigraft seroma in a clinical setting can be challenging, and the differential diagnoses encompass a wide range of conditions, including infection, pseudoaneurysm, postoperative hematoma, and lymphatic fluid collection [2]. In the presented case, the diagnosis was initially confused and considered to be surgical site infection. The possibility of lymphatic fistula was not considered due to the smooth decrease in fluid drainage from chest tubes after redo-AVR without any abnormal color change. However, in retrospect, it would have been beneficial to perform a leaking fluid analysis to fully exclude the possibility of it [3].

Since triplex graft is a relatively new development involving the use of an artificial material [4], there is limited information available on perigraft seroma [5]. The graft consists of three layers: an internal layer made of knitted material, a middle layer made of non-biodegradable elastomer, and an outer layer made of woven material. It is generally thought that the impermeability of the mid-layer should prevent bleeding and seroma [6]. It does not require pre-clotting and has been shown to suppress immunological reactions. In theory, it does not pose a risk of seroma development due to ultrafiltration [7]. However, it is important to note that these advantages may be diminished when used as a patch in a Triplex graft.

While a much more invasive redo graft replacement could be prevented in the case presented, long-standing percutaneous drainage of perigraft seroma carries with it the potential risks of skin erosion, continuous pain, and retrograde infection. To mitigate the risk of infection caused by normal bacterial flora of the skin, it is emphasized that the patient must adhere to a regular and meticulous daily disinfection procedure after hospital discharge.

In summary, perigraft seroma was resolved without implanted Triplex graft removal after 14 months of percutaneous fluid drainage following several ineffective surgical treatments. The diagnosis was initially confusing and was considered to be surgical site infection. The structural characteristics of the graft, including its layered design with a non-biodegradable mid-layer, were deemed inadequate to prevent the occurrence of perigraft seroma.

## Data Availability

There are no additional data to disclose.

## Abbreviations

AVR: Aortic valve replacement
CT: Computed tomography
ePTFE: Expanded polytetrafluoroethylene
